# Cryptococcal meningoencephalitis in patients with mantle cell lymphoma on ibrutinib

**DOI:** 10.3332/ecancer.2018.836

**Published:** 2018-05-17

**Authors:** Kai Sun, Saro Kasparian, Swaminathan Iyer, Sai Ravi Pingali

**Affiliations:** 1 Department of Medicine, Houston Methodist Hospital, Houston, TX 77030, USA; 2 Department of Hematology, Houston Methodist Hospital, Houston, TX 77030, USA

**Keywords:** cryptococcal meningoencephalitis, mental cell lymphoma, ibrutinib

## Abstract

Ibrutinib, a Bruton’s tyrosine kinase inhibitor, has been increasingly widely used in relapsed and refractory mantle cell lymphoma (MCL) and chronic lymphocytic leukaemia [1, 2]. With its use becoming more common, there have been emerging case reports of opportunistic infections like cryptococcal infections [3–8]. These infections in patients receiving ibrutinib were mostly reported in patients with chronic lymphocytic leukaemia, who have poor immune reconstitution. Here, we report two cases of cryptococcal meningoencephalitis in patients with MCL on ibrutinib.

## Case 1

A 70-year-old male with a past medical history of type II diabetes and primary refractory stage IV B mantle cell lymphoma (MCL) presented with altered mental status for 2 weeks. The patient was treated with three cycles of R-CHOP (rituximab, cyclophosphamide, doxorubicin, vincristine and prednisone) without response. Following that, he was treated with bortezomib and rituximab with partial response. Five months prior to presentation, the chemotherapy regimen was changed to ibrutinib 560 mg daily with rituximab 375 mg weekly for 4 weeks followed by rituximab monthly. Five months after he had been on ibrutinib and rituximab, he presented with a 2-week history of altered mental status, visual hallucination that was worse at night and headache. He denied fevers, chills, nausea, vomiting or diarrhoea. Laboratory results revealed sodium of 126 mEq/L, normal white blood cell (WBC) of 5.15 × 10^9^/L with 81% neutrophils and 12% lymphocytes, normocytic normochromic anaemia with red blood cell (RBC) of 2.79, haemoglobin of 9.2 g/dL and thrombocytopenia with platelet of 95 × 10^9^/L. Magnetic resonance imaging (MRI) of the brain with and without contrast revealed two punctate foci of enhancement in the posterior left basal ganglia measuring 2–3 mm and 1.5 mm, respectively. His altered mental status was initially attributed to hyponatraemia, and the patient was started on a fluid restricted diet, sodium tablet and later tolvaptan. However, his symptoms, including headache and visual hallucinations, didn’t improve and the patient started having low grade fevers. Lumbar puncture was done 1 week after hospitalisation which showed slightly hazy pale yellow cerebral spinal fluids (CSFs), decreased glucose of 36 mg/dL, elevated protein of 159 mg/dL, 571 WBC with 41% lymphocytes and 59% neutrophils, 1107 RBC. CSF cryptococcal antigen titre was 1:2 and serum cryptococcal antigen titre was 1:64. No growth on CSF fungal culture. Ibrutinib was discontinued. The patient was started on amphotericin 3 mg/kg/day and flucytosine 1500 mg every 8 hours for 14 days with improvement of symptoms and transitioned to oral fluconazole 400 mg twice a day. Repeated serum cryptococcal antigen titres on follow-up visits kept trending down till nondetectable. He was continued on fluconazole and was started on lenalidomide and rituximab later for the MCL.

## Case 2

A 78-year-old male with a past medical history of stage IV MCL presented with imbalance, headache behind the eyes and double vision that progressively got worse within a period of several months. The patient developed fevers later after admission. His stage IV MCL was diagnosed in 2006 with bone marrow involvement and was heavily treated with multiple chemotherapies, including R-CHOP, bortezomib and rituximab, bendamustine and rituximab and Bexxar and lenalidomide from 2006 to 2013. Ibrutinib 540 mg daily was initiated after his third relapse 2 years prior to his presentation and he had remained in complete remission. Laboratory findings revealed leukopenia with WBC of 1.51 × 10^9^/L with 68.9% neutrophils and 17.7% lymphocytes, anaemia with haemoglobin of 9.5 g/dL, thrombocytopenia with platelet of 89 × 10^9^/L. MRI brain with and without contrast showed vasogenic oedema in the right parieto-occipital region with overlying gyral thickening and gyriform enhancement with mild regional mass effect. Patient subsequently underwent lumbar puncture with opening pressure of 21-cm water. CSF was clear with normal glucose of 42 mg/dL, elevated protein of 61 mg/dL, 18 WBC with 78% lymphocytes, 19% monocytes and 3% neutrophils and 12 RBC. CSF cryptococcal antigen was positive while fungal smear and cultures showed no growth. Ibrutinib was discontinued. He was started on amphotericin and flucytosine for 14 days with an improvement of his symptoms and was continued on oral fluconazole. Repeated lumbar puncture 2 weeks after the initiation of treatment showed normal glucose of 49 mg/dL, normal protein of 41 mg/dL, 4 WBC and 1 RBC. The CSF cryptococcal antigen became negative. The patient remained in remission off ibrutinib or other medications for MCL until 4 months later when he was found to have therapy-related myelodysplastic syndrome which later transformed to acute myeloid leukaemia.

## Discussion

Cryptococcal infection is an opportunistic fungal infection with a predilection to the lungs and central nervous system [9, 10]. The majority of cases of cryptococcal infection were *Cryptococcal neoformans* and most often happened in patients with human immunodeficiency virus (HIV) infection. The cases that occurred in non-HIV-infected individuals were transplant recipients, patients who are receiving immunosuppressive agents such as glucocorticosteroids and cytotoxic chemotherapy and patients with innate immunologic problems [11]. It was found that cell immunity plays an essential role in preventing and controlling the cryptococcal infections [9, 10].

Bruton’s tyrosine kinase (BTK) is a cytoplastic tyrosine kinase of the Tec family that is essential for B-cell receptor signalling and B-cell survival [12]. Ibrutinib works as an orally active inhibitor of BTK that covalently binds to cysteine Cys-481 of BTK and irreversibly inactivates the kinase [12, 13]. Ibrutinib has shown its effect in treating chronic lymphocytic leukaemia (CLL), MCL and other B-cell lymphoproliferative diseases [1, 2]. However, emerging cases of cryptococcal infections in patients on ibrutinib have been reported, most of which were patients with CLL.

Okamoto *et al* [8] reported a case of disseminated cryptococcosis in a patient with CLL after being on ibrutinib for 1 month. Messina *et al* [7] later reported two cases of cryptococcal infection involving central nervous system in a patient with indolent lymphoma and one patient with CLL. Both patients presented shortly after they were started on ibrutinib (3 weeks and 1 month, respectively). Another retrospective study done by Varughese *et al* [14] which looked at serious infections in the patients receiving ibrutinib found that 16/378 developed opportunistic fungal infections. Ibrutinib was started within 1 year in most of the patients. Ajam *et al* [3] reported a primary cutaneous case in a patient with relapsed CLL. A large retrospective study performed by Rogers *et al* [15] which looked at the opportunistic infections in the patients on ibrutinib revealed that one CLL patient out of 566 patients with haematologic malignancies had cryptococcal infection.

Cases of cryptococcal infection in the MCL patients on ibrutinib have been scarcely reported. It was observed that ibrutinib inhibits antigen receptor signalling in B cells not T cells [16]. As mentioned above, cell immunity plays an important role in cryptococcal infection and should be relatively intact in the MCL patients who are on cytotoxic chemotherapies. One mechanism of the infection might be ibrutinib’s indirect effect on cell immunity. BTK has been demonstrated to participate in the regulation of nitric oxide induction and bactericidal functions in macrophages [17] which interact with helper cells and participate in cell immunity. Ibrutinib might indirectly affect cell immunity by inhibiting cells that interact with T cell. Szymczak *et al* [18] found that X-linked immunodeficiency mice were not able to contain cryptococcus infection in the lungs due to reduced uptake by macrophages. The inhibition of macrophages by ibrutinib could be another mechanism.

## Conclusions

The majority of previous case reports about cryptococcal infections in patients with haematologic malignancies on ibrutinib were patients with CLL. In addition, the onset of symptoms was shortly after they were started on ibrutinib. Both of our patients had MCL which was not known to be associated with cell immunity deficiency, and cryptococcal infections were scarcely reported in MCL patients on ibrutinib. They had been on ibrutinib for 5 months and 2 years, respectively, before they presented with symptoms, which also distinguishes our report from previous case reports. As ibrutinib gains more popularity in MCL, it is important for physicians to stay vigilant to keep cryptococcal infection in the differentials even after patients have been on this medication for a relatively long period of time so that timely diagnosis and treatment can be implemented.

## Conflicts of interest

The authors declare that they have no conflicts of interest.
